# The Revised Fibromyalgia Impact Questionnaire (FIQR): validation and psychometric properties

**DOI:** 10.1186/ar2783

**Published:** 2009-08-10

**Authors:** Robert M Bennett, Ronald Friend, Kim D Jones, Rachel Ward, Bobby K Han, Rebecca L Ross

**Affiliations:** 1 Fibromyalgia Research Unit, Oregon Health & Science University, 3455 SW Veterans Road, Portland, OR 97239, USA; 2 Department of Psychology, Stony Brook University, Stony Brook, NY 11794-2500, USA; 3 Physicians Building Group, 1234 Commercial Street SE, Salem, OR 97302, USA

## Abstract

**Introduction:**

The Fibromyalgia Impact Questionnaire (FIQ) is a commonly used instrument in the evaluation of fibromyalgia (FM) patients. Over the last 18 years, since the publication of the original FIQ, several deficiencies have become apparent and the cumbersome scoring algorithm has been a barrier to widespread clinical use. The aim of this paper is to describe and validate a revised version of the FIQ: the FIQR.

**Methods:**

The FIQR was developed in response to known deficiencies of the FIQ with the help of a patient focus group. The FIQR has the same 3 domains as the FIQ (that is, function, overall impact and symptoms). It differs from the FIQ in having modified function questions and the inclusion of questions on memory, tenderness, balance and environmental sensitivity. All questions are graded on a 0–10 numeric scale. The FIQR was administered online and the results were compared to the same patient's online responses to the 36-Item Short Form Health Survey (SF-36) and the original FIQ.

**Results:**

The FIQR was completed online by 202 FM patients, 51 rheumatoid arthritis (RA) or systemic lupus erythematosus (SLE) patients (31 RA and 20 SLE), 11 patients with major depressive disorder (MDD) and 213 healthy controls (HC). The mean total FIQR score was 56.6 ± 19.9 compared to a total FIQ score of 60.6 ± 17.8 (*P *< 0.03). The total scores of the FIQR and FIQ were closely correlated (*r *= 0.88, *P *< 0.001). Each of the 3 domains of the FIQR correlated well with the 3 related FIQ domains (*r *= 0.69 to 0.88, *P *< 0.01). The FIQR showed good correlation with comparable domains in the SF-36, with a multiple regression analysis showing that the three FIQR domain scores predicted the 8 SF-36 subscale scores. The FIQR had good discriminant ability between FM and the 3 other groups; total FIQR scores were HC (12.1 ± 11.6), RA/SLE (28.6 ± 21.2) and MDD (17.3 ± 11.8). The patient completion time was 1.3 minutes; scoring took about 1 minute.

**Conclusions:**

The FIQR is an updated version of the FIQ that has good psychometric properties, can be completed in less than 2 minutes and is easy to score. It has scoring characteristics comparable to the original FIQ, making it possible to compare past FIQ results with future FIQR results.

## Introduction

The Fibromyalgia Impact Questionnaire (FIQ) was developed in the late 1980s and was first published in 1991 [1], with minor revisions in 1997 and 2002 [2]. It has subsequently become one of the most frequently used tools in the evaluation of fibromyalgia (FM) patients [2-4], being cited in over 300 articles and translated into 14 languages. Over the 18 years since its publication, problems in regard to some aspects of its content and rather cumbersome scoring algorithm have become apparent [4-6]. The original questionnaire used a visual analog scale (VAS) that required patients to slash a 100-mm line and was scored with a ruler. The scoring was further complicated by the need to reverse scores in one question and the use of constants to convert the first 13 questions to a standardized scale of 0 to 10. The functional questions in the first part of the FIQ were originally intended for women living in reasonably affluent countries and assumed the possession of a car, a vacuum cleaner, and a washing machine. Moreover, questions that now are considered relevant, such as dyscognition, tenderness, balance, and environmental sensitivity, were not part of the original FIQ. With these issues in mind, we have developed an online and paper-equivalent version of the questionnaire: the Revised Fibromyalgia Impact Questionnaire (FIQR) (Additional data file 1). The FIQR attempts to address the limitations of the FIQ while retaining the essential properties of the original instrument.

## Materials and methods

### Focus group testing

A draft version of the new questionnaire was constructed by RMB and tested in a focus group of 10 female patients with FM (age 58 ± 5.4 years, age range 51 to 68 years; FM duration 22 ± 12.7 years, duration range 3 to 40 years). The focus group was guided by RMB with the assistance of KDJ, RLR, and RW. It was conducted in a manner that encouraged the free interchange of ideas. The revised questions were based on previous experience with the FIQ and patients' evaluation of important symptoms as recorded in OMERACT 8 (Outcome Measures in Rheumatology) [7], International Classification of Functioning, Disability, and Health (ICF) guidelines [8], and patient surveys from the US [9] and Germany [10]. The draft modifications of the original FIQ were sixfold: (a) perform all scoring with 11 boxes (scaled 0 to 10) instead of a mixture of Likert measurements and VAS measurements; (b) modify the functional questions (numbers 1 to 11 in the original FIQ); (c) modify the two impact questions (numbers 12 and 13 in the original FIQ); (d) expand the symptom questions (numbers 14 to 20 in the original FIQ) to include tenderness, dyscognition, balance, and environmental sensitivity; (e) simplify the scoring algorithm; and (f) modify the weighting of the three domains (function, overall impact, and symptoms) to give more weight to function. The proceedings were digitally recorded and transcribed by RW. Following a discussion among patients and investigators, modifications were made to the draft version of the FIQR and agreement was reached on the final version of the FIQR (Table 1). For instance, an original FIQ question regarding 'walking several blocks' was modified by the focus group to 'walk continuously for 20 minutes' as the concept of a block varies from city to city and country to country. The entirely new question, 'sit in a chair for 45 minutes', arose out of a discussion on problems associated with pain and immobility. As it was intended to conduct the validation of the FIQR online, the use of this collection method and the validity of using 11 boxes rather than 0- to 100-mm VASs were compared between the following five versions of the questionnaires that were completed by the focus group: (a) the original paper version of the FIQ (FIQ-P), (b) an online version of the FIQ (FIQ-OL), (c) a paper version of the FIQR using 11 boxes scaled 0 to 10 (FIQR-P), (d) a paper version of the FIQR using a 100-mm VAS scoring (FIQR-P VAS), and (e) an online version of the FIQR (FIQR-OL). The online versions of the FIQR and FIQ were completed 4 weeks after completion of the paper versions.

**Table 1:** The Revised Fibromyalgia Impact Questionnaire

Domain 1 directions: For each of the following nine questions, check the one box that best indicates how much your fibromyalgia made it difficult to do each of the following activities over the past 7 days:	
Brush or comb your hair	No difficulty □ □ □ □ □ □ □ □ □ □ □ Very difficult
Walk continuously for 20 minutes	No difficulty □ □ □ □ □ □ □ □ □ □ □ Very difficult
Prepare a homemade meal	No difficulty □ □ □ □ □ □ □ □ □ □ □ Very difficult
Vacuum, scrub, or sweep floors	No difficulty □ □ □ □ □ □ □ □ □ □ □ Very difficult
Lift and carry a bag full of groceries	No difficulty □ □ □ □ □ □ □ □ □ □ □ Very difficult
Climb one flight of stairs	No difficulty □ □ □ □ □ □ □ □ □ □ □ Very difficult
Change bed sheets	No difficulty □ □ □ □ □ □ □ □ □ □ □ Very difficult
Sit in a chair for 45 minutes	No difficulty □ □ □ □ □ □ □ □ □ □ □ Very difficult
Go shopping for groceries	No difficulty □ □ □ □ □ □ □ □ □ □ □ Very difficult
	
Domain 2 directions: For each of the following two questions, check the one box that best describes the overall impact of your fibromyalgia over the past 7 days:	

Fibromyalgia prevented me from accomplishing goals for the week	Never □ □ □ □ □ □ □ □ □ □ □ Always
I was completely overwhelmed by my fibromyalgia symptoms	Never □ □ □ □ □ □ □ □ □ □ □ Always
	
Domain 3 directions: For each of the following 10 questions, check the one box that best indicates the intensity of your fibromyalgia symptoms over the past 7 days:	

Please rate your level of pain	No pain □ □ □ □ □ □ □ □ □ □ □ Unbearable pain
Please rate your level of energy	Lots of energy □ □ □ □ □ □ □ □ □ □ □ No energy
Please rate your level of stiffness	No stiffness □ □ □ □ □ □ □ □ □ □ □ Severe stiffness
Please rate the quality of your sleep	Awoke rested □ □ □ □ □ □ □ □ □ □ □ Awoke very tired
Please rate your level of depression	No depression □ □ □ □ □ □ □ □ □ □ □ Very depressed
Please rate your level of memory problems	Good memory □ □ □ □ □ □ □ □ □ □ □ Very poor memory
Please rate your level of anxiety	Not anxious □ □ □ □ □ □ □ □ □ □ □ Very anxious
Please rate your level of tenderness to touch	No tenderness □ □ □ □ □ □ □ □ □ □ □ Very tender
Please rate your level of balance problems	No imbalance □ □ □ □ □ □ □ □ □ □ □ Severe imbalance
Please rate your level of sensitivity to loud noises, bright lights, odors, and cold	No sensitivity □ □ □ □ □ □ □ □ □ □ □ Extreme sensitivity

Scoring: Step 1. Sum the scores for each of the three domains (function, overall, and symptoms). Step 2. Divide domain 1 score by three, divide domain 2 score by one (that is, it is unchanged), and divide domain score 3 by two. Step 3. Add the three resulting domain scores to obtain the total Revised Fibromyalgia Impact Questionnaire score.

### The Revised Fibromyalgia Impact Questionnaire and its scoring

The revised FIQ (the FIQR) has 21 individual questions (Table 1). All questions are based on an 11-point numeric rating scale of 0 to 10, with 10 being 'worst'. As in the FIQ, all questions are framed in the context of the past 7 days. Following the convention used in the FIQ, the FIQR is divided into three linked sets of domains: (a) 'function' (contains 9 questions versus 11 in the FIQ), (b) 'overall impact' (contains 2 questions, as in the FIQ) but the questions now relate to the overall impact of FM on functioning and the overall impact symptom severity, and (c) 'symptoms' (contains 10 questions versus 7 in the FIQ); one original FIQ symptom was dropped: 'When you worked, how much did pain or other symptoms of your fibromyalgia interfere with your ability to do your work, including housework?' The symptom domain contains four new questions relating to memory, tenderness, balance, and environmental sensitivity (to loud noises, bright lights, odors, and cold temperatures). The 'time' dimension is the same as the FIQ; that is, all questions relate to the impact of FM over the course of the past 7 days. The scoring of the FIQR is much simpler than the FIQ: namely, the summed score for function (range 0 to 90) is divided by 3, the summed score for overall impact (range 0 to 20) is not changed, and the summed score for symptoms (range 0 to 100) is divided by 2. The total FIQR is the sum of the three modified domain scores. The weighting of these three domains is different from the FIQ in that 30% of the total score is ascribed to 'function' as opposed to 10% in the FIQ, 50% is ascribed to 'symptoms' as opposed to 70% in the FIQ, and 'overall impact' remains the same as the FIQ at 20%. The total maximal score of the FIQR remains the same as the FIQ, namely 100.

### Subjects

All of the FM subjects were patients diagnosed within the previous 5 years with FM as defined by the American College of Rheumatology (ACR) [11]. They had indicated that they were interested in being contacted in regard to FM research studies. The patients with either rheumatoid arthritis or systemic lupus erythematosus (RA/SLE) were all patients being currently treated and followed in the clinical practice of BKH; patients with coexisting FM were excluded initially by prescreening the patient charts for a diagnosis of FM and then re-evaluating each subject prior to entry into the study. The patients with major depressive disorder (MDD) were all patients being currently treated and followed in the clinical practice of RLR; patients with coexisting FM were excluded as above. The healthy control group consisted of coworkers, friends, and relatives; they were requested to email the questionnaire link to acquaintances whom they considered to be in good health. All participants completed online informed consent, and the study was conducted in accordance with the Declaration of Helsinki.

### Data collection

The questionnaires were formatted for use on Survey Monkey (Portland, OR, USA), a commercial online survey technology. In addition to the FIQR, the original questionnaire (FIQ) and the 36-Item Short Form Health Survey (SF-36) (Rand Corporation, Santa Monica, CA, USA) were posted on the Survey Monkey site for the FM subjects. The SF-36 is a widely used generic instrument that measures health-related quality of life [12] and has a well-documented use in the evaluation of FM patients [13,14]. The online site for the healthy controls and RA, SLE, and MDD subjects did not contain the FIQ or SF-36 questionnaire. The questionnaire for healthy controls and RA, SLE, and MDD patients differed from the questionnaire for FM patients in that the term 'health issues' was substituted throughout the questionnaire for 'fibromyalgia' (this questionnaire, the SIQR, is available in the online version of this article; Additional data file 2). To ascertain that FM subjects still had widespread pain and that the healthy controls and RA, SLE, and MDD patients did not have widespread pain, the questionnaire contained a 'yes/no' item as to the body areas in which they currently had pain. This item contained 24 separate locations: left shoulder, right shoulder, left jaw, right jaw, left upper back, right upper back, left arm, right arm, left hand, right hand, left lower back, right lower back, left hip, right hip, left thigh, right thigh, left knee, right knee, left foot, right foot, mid upper back, mid lower back, front of chest, and neck.

The survey was sent out to 659 FM patients in August 2008, and 208 responded within 2 weeks (a response rate of 32%). After approximately 200 FM subjects had completed the questionnaire, the results were downloaded from the Survey Monkey server into Excel spreadsheets (Microsoft Corporation, Redmond, WA, USA) and the survey was closed to further participation for the FM patients. The RA/SLE and the MDD sites were kept open for about 3 months as it was challenging to find RA, SLE, and MDD patients who did not have widespread pain. The FIQR scoring algorithm was processed on the Excel spreadsheet and then transferred to STATISTICA statistical software (StatSoft, Inc., Tulsa, OK, USA) for the statistical analyses. As a check on data entry and scoring, the Excel spreadsheet was also loaded into version 14 of SPSS statistical software (SPSS Inc., Chicago, IL, USA) and the scoring algorithm was entered into SPSS syntax. Correlation and verification of the STATISTICA data and results were performed by RW and KDJ.

### Data analysis

All data were analyzed in STATISTICA (version 8). Item analysis and questionnaire properties, including domain characteristics, were evaluated using basic statistics, reliability item analysis, and Cronbach alpha. Group comparisons on the mean total FIQR scores and individual FIQR items used one-way analysis of variance (ANOVA) and multivariate ANOVA for single and multiple dependent variables, respectively, with Tukey honestly significantly differences (HSD) *post hoc *analyses for unequal sample sizes comparing the significance of specific means. FIQR validity was established using correlational analyses between FIQR, FIQ, and SF-36 items and domains. Correlations were assessed using Pearson's product moment correlation coefficient (*r*). Multiple regression was used to establish convergent and discriminant validity. The three FIQR domains were entered simultaneously as predictors to determine their combined contribution of variance in SF-36 subscales. Standardized regression coefficients (β) were calculated to evaluate the unique contribution of the three FIQR domains to the SF-36 subscales, and the partial correlation coefficients (pr) were calculated to determine the correlation of each of the three FIQR domains to the SF-36 subscales after controlling for the other two domains.

## Results

### Focus group

The focus group tested the relatedness of two versions of the FIQ (FIQ-P and FIQ-OL) versus three versions of the FIQR (FIQR-P, FIQR-P VAS, and FIQR-OL). Converting the FIQ to an online questionnaire did not significantly affect its total mean scores (59.8 versus 61.8) (Table 2). The use of 11 boxes rather than 0- to 100-mm VASs did not significantly affect the total mean scores of the paper version of the FIQR (56.4 versus 57.6). Finally, the online version of the FIQR had a total score similar to that of the paper version of the FIQ (59.7 versus 59.8), with a correlation coefficient of 0.83 (*P *< 0.005). These results provided some confidence that an online version of the FIQR, with 11-box scoring (0 to 10), would probably have operating characteristics similar to those of the well-validated paper version of the original questionnaire (FIQ) that uses VAS scoring. As the online versions were completed 4 weeks after the paper versions, the similarity of scoring and correlations of the respective paper and online scores provide some evidence for test-retest reliability.

**Table 2:** Focus group total scores and correlations of the various versions of the Fibromyalgia Impact Questionnaire and the Revised Fibromyalgia Impact Questionnaire

	Mean ± SD	FIQ-P	FIQR-P	FIQR-P VAS	FIQ-OL	FIQR-OL
FIQ-P	59.8 ± 20.9	-	0.93	0.94	0.91	0.83
FIQR-P	57.6 ± 26.3	0.93	-	0.99	0.94	0.89
FIQR-P VAS	56.4 ± 27.6	0.94	0.99	-	0.94	0.88
FIQ-OL	61.8 ± 21.2	0.91	0.94	0.94	-	0.95
FIQR-OL	59.7 ± 24.9	0.83	0.89	0.88	0.95	^-^

All correlations were significant at *P *< 0.001. FIQ-OL, an online version of the Fibromyalgia Impact Questionnaire; FIQ-P, the original paper version of the Fibromyalgia Impact Questionnaire; FIQR-OL, an online version of the Revised Fibromyalgia Impact Questionnaire; FIQR-P, a paper version of the Revised Fibromyalgia Impact Questionnaire using 11 boxes scaled 0 to 10; FIQR-P VAS, a paper version of the Revised Fibromyalgia Impact Questionnaire using a 100-mm visual analog scale scoring instead of 11 boxes; SD, standard deviation.

The focus group also completed the SF-36 to compare ease of use and timing. During the focus group meeting, the FM patients contributed to the face validity of the final version by suggesting modifications in wording. For instance, the original FIQ question regarding 'walking several blocks' was reworded to 'walk continuously for 10 minutes', 'climb stairs' was modified to 'climb one flight of stairs', 'make beds' was modified to 'change bed sheets', 'do shopping' was modified to 'go shopping for groceries', and 'vacuum a rug' was modified to 'vacuum, scrub, or sweep floors'. The focus group also suggested two new questions: 'brush or comb your hair' and 'sit in a chair for 45 minutes'. The 'brush or comb hair' was to be the first question in the 'function' set as it is usually the least problematic activity for FM patients and would set the difficulty level for the following eight questions. The results from this focus group helped to provide some confidence that it would be feasible to use online data collection in that converting the 0- to 100-mm VASs and the Likert questions from the FIQ to an 11-point numeric rating scale (0 to 10) would not appreciably compromise the comparison of the FIQR with the FIQ. Patient completion times for the paper versions of the original FIQ, the FIQR, and the SF-36 were 2.1 ± 0.03 minutes, 1.3 ± 0.02 minutes, and 4.1 ± 0.04 minutes, respectively. The time taken for investigator scoring of the FIQR was approximately 1 minute.

### Analysis of Revised Fibromyalgia Impact Questionnaire properties

A total of 208 FM patients completed the online questionnaires (FIQR, FIQ, and SF-36). There were 21 FM subjects who had fewer than 10 pain locations; on further review of their pain distribution, 2 subjects did not meet the ACR criteria for widespread pain and were removed from the survey. Another four questionnaires were incomplete. Thus, 202 completed questionnaires were available for analysis. The demographics of the FM patients and the other three groups are shown in Table 3. The groups differed in age, F(3,473) = 492.12 (*P *< 0.001), with FM patients being 8 years older than healthy controls (*P *< 0.001). As expected, the four groups differed substantially in regard to pain locations, F(3,473) = 492.12 (*P *<0.001), with FM patients having many more pain locations than the other three groups (all *P *< 0.001). The total FIQR scores in the RA (n = 31) and SLE (n = 20) patients were similar and not significantly different (RA: 28 ± 21.0 and SLE: 30 ± 22.5, *P *= 0.74). Hence, the two groups were merged into a single group (RA/SLE) as the intent was to compare an inflammatory rheumatic disease group with FM. The healthy group had fewer pain locations than the RA/SLE groups (*P *< 0.001), while the MMD group did not differ from either the healthy controls (*P *= 0.55) or the RA/SLE (*P *= 0.29).

**Table 3:** Demographics of fibromyalgia patients and other groups

	Fibromyalgia	RA/SLE	Major depression^a^	Healthy controls
Number analyzed	202	51	11	213
Age, years	51 ± 10.5	49 ± 13.1^a^	46 ± 11.4^b^	43 ± 14.0^c^
Gender ratio, female/male	16/1	ND	5 ± 1	13 ± 1
Number of pain locations	16 ± 4.9	7.0 ± 4.4	4.0 ± 2.5	1.6 ± 2.3

In comparison with the fibromyalgia patients: ^a^*P *= 0.25; ^b^*P *= 0.13; ^c^*P *< 0.001. ND, not determined; RA/SLE, rheumatoid arthritis/systemic lupus erythematosus.

**Figure 1:**
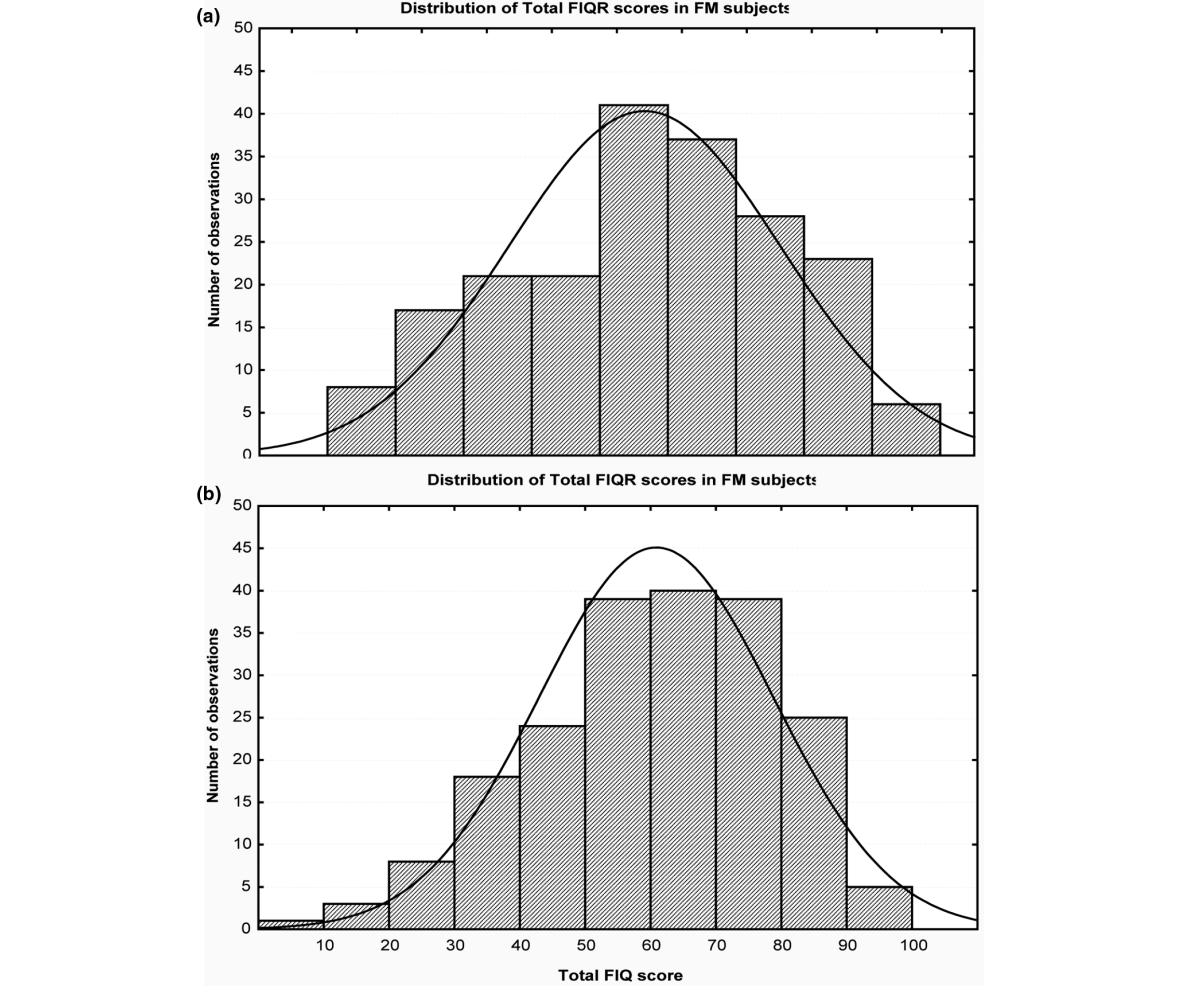
Histograms of FIQ and FIQR showing distributions of total scores. **(a) **The distribution profile of the total Revised Fibromyalgia Impact Questionnaire (FIQR) scores in 202 fibromyalgia (FM) patients. **(b) **The distribution profile of the total Fibromyalgia Impact Questionnaire (FIQ) scores. There is a slight negative skewness for both distributions. The FIQR Shapiro-Wilk skewness coefficient (W) is 0.978, and the FIQ Shapiro-Wilk skewness coefficient (W) is 0.980.

The patient FIQR scores, though appearing to be normally distributed, were negatively skewed (Shapiro-Wilk W = 0.978, *P *= 0.003), slightly favoring the more severe cases (Figure 1a). This FIQR distribution was nearly identical to the distribution of FIQ scores (Figure 1b), which were also slightly negatively skewed (Shapiro-Wilk W = 0.980, *P *= 0.006). The mean FIQR total score was 56.6 ± 19.9, with a median score of 58 (95% confidence interval [CI] 53.8, 59.4) (Table 4). The mean FIQ total score was 60.6 ± 17.9, with a median score of 61.9 (95% CI 58.1, 63.0). There were only 12 FM males compared with 190 FM females, and the respective total FIQR scores were 53.2 ± 20.4 and 56.8 ± 20.0 (*P *= 0.55). Higher scores are indicative of greater dysfunction or symptom severity, and the FIQR sleep quality question had the highest score (7.61 ± 2.4), followed by tenderness to touch (6.86 ± 2.5), energy level (6.80 ± 2.4), stiffness (6.72 ± 2.2), environmental sensitivity (6.19 ± 2.9), and pain (6.01 ± 2.1). As expected, 'difficulty with combing hair' had the lowest score (2.42 ± 2.6), but seven patients had scores of at least 8 on this question. The Cronbach alpha for the FIQR was 0.95, with item-total correlations ranging from 0.56 to 0.93. The item-total correlations for the four new items were 0.69 for memory, 0.56 for tenderness, 0.65 for balance, and 0.57 for sensitivity, strongly justifying their inclusion as part of the FIQR.

**Table 4:** Revised Fibromyalgia Impact Questionnaire question values in 202 patients with fibromyalgia

	Mean	Median	One SD	-95% CI	+95% CI	Correlation with total FIQR score	Score range
Comb hair	2.4	2	2.6	2.1	2.8	0.62	0–10
Walk for 20 minutes	5.7	6	3.5	5.3	6.2	0.72	0–10
Prepare a meal	4.3	4	3.2	3.9	4.7	0.77	0–10
Clean floors	6.5	7	3.0	6.1	6.9	0.75	0–10
Carry a bag of groceries	5.6	6	3.2	5.2	6.0	0.76	0–10
Climb a flight of stairs	5.6	5	3.3	5.1	6.0	0.80	0–10
Change bed sheets	5.5	6	3.2	5.1	6.0	0.79	0–10
Sit for 45 minutes	5.6	6	3.2	5.1	6.0	0.59	0–10
Go shopping for groceries	5.6	6	3.2	5.2	6.1	0.81	0–10
FIQR function	15.6	15	7.7	14.5	16.7	0.90	0–30
Can't achieve goals	5.7	6	2.9	5.3	6.1	0.85	0–10
Feel overwhelmed	5.2	5	2.9	4.8	5.6	0.86	0–10
FIQR overall	11.0	11	5.4	10.2	11.7	0.91	0–20
Pain rating	6.0	6	2.1	5.7	6.3	0.72	0–10
Energy rating	6.8	7	2.4	6.5	7.1	0.69	0–10
Stiffness rating	6.7	7	2.9	6.4	7.0	0.62	0–10
Sleep quality	7.6	8	2.4	7.3	7.9	0.57	0–10
Depression level	4.6	5	2.9	4.2	5.0	0.60	0–10
Memory problems	5.9	6	2.6	5.6	6.3	0.69	0–10
Anxiety level	4.5	5	3.1	4.0	4.9	0.62	0–10
Tenderness level	6.9	7	2.5	6.6	7.2	0.56	0–10
Balance problems	4.8	5	2.9	4.4	5.2	0.65	0–10
Environmental sensitivity	6.2	7	2.9	5.8	6.6	0.57	0–10
FIQR symptoms	30.0	31	8.8	28.8	31.2	0.93	0–50
FIQR total	56.6	58	20.0	53.8	59.4	-	0–100

CI, confidence interval; FIQR, Revised Fibromyalgia Impact Questionnaire; SD, standard deviation.

The goal of giving more weight to function in the FIQR appears to have been successful. Table 5 presents the new weighting for the three FIQR domains contrasted with the original weighting in the FIQ (columns 2 and 4). Columns 3 and 5 present the observed (actual) means for the FIQR and FIQ with the contribution of each domain mean score presented as a percentage of the total scores. As can be seen, the 'imbalance' observed in the FIQ between function and symptom (7% and 74%) has been markedly improved in the FIQR (28% and 53%), approximating the new weighting given to scoring the FIQR (30% and 50%). The contribution of overall impact to total score (19% in FIQ and 19% in FIQR) also approximates the 20% weighting given in each scale. While the new weighting for the FIQR seems to have been successful, there was a significant 3.99-point difference in the total mean scores (*P *< 0.03). This may be due to the change in weighting reflected by a smaller increase in function scores (+11.31) relative to a greater decrease in symptom scores (-14.85), as shown in column 6, and/or because of other changes and additions to the questions in the FIQR.

**Table 5:** Comparison of Fibromyalgia Impact Questionnaire and Revised Fibromyalgia Impact Questionnaire weighting on actual and achieved domain scores

	FIQ		FIQR		Change
			
	Given weight	Achieved weight	Given weight	Achieved weight	
Function	10%	4.30 (7%)	30%	15.61 (28%)	+11.31
Overall impact	20%	11.42 (19%)	20%	10.97 (19%)	-0.45
Symptoms	70%	44.85 (74%)	50%	30.00 (53%)	-14.85
Total	60.57 (100%)		56.58 (100%)		-3.99

This analysis shows that the weighting of the Revised Fibromyalgia Impact Questionnaire (FIQR) closely approximates the given weight. The 'imbalance' observed in the Fibromyalgia Impact Questionnaire (FIQ) between function and symptom (7% and 74%) has been markedly improved in the FIQR (28% and 53%).

Convergent validity was assessed by comparing the FIQR to both the SF-36 and the FIQ. Note that all of the correlations of the FIQ with the SF-36 are negative due to the fact that higher scores on the SF-36 relate to being healthier. The SF-36 subscale scores in the FM patients were physical functioning 39.8 ± 24.4, physical role 13.5 ± 27.1, emotional role 39.1 ± 43.0, vitality 17.6 ± 14.3, emotional health 57.4 ± 20.2, social functioning 43.6 ± 32.5, bodily pain 33.9 ± 18.3, and general health 38.2 ± 21.3. These SF-36 subscale scores were similar to our previous findings [15] and a review of the literature [13], helping to confirm that the FM population in this study was comparable to most other studies. In general, the three domains of the FIQR and the individual questions correlated most closely with the corresponding subscales on the SF-36 (Table 6). For instance, the FIQR total score correlated best with SF-36 physical functioning and pain subscales (*r *= -0.71 and -0.69), the FIQR function domain correlated best with SF-36 physical functioning and pain subscales (*r *= -0.80 and -0.60), the FIQR overall impact domain correlated best with the SF-36 physical functioning and pain subscales (*r *= -0.60 and -0.64), and the FIQR symptoms domain closely correlated with all of the SF-36 subscales (*r *= -0.43 to -0.66). When individual questions were looked at, the FIQR pain correlated best with SF-36 pain (*r *= -0.66), and FIQR anxiety and depression correlated best with the SF-36 mental health subscale (*r *= -0.72 and -0.63).

**Table 6:** Pearson correlations of the Revised Fibromyalgia Impact Questionnaire with subscales of the 36-Item Short Form Health Survey

	Physical functioning SF-36	Physical role SF-36	Emotional role SF-36	Vitality (energy) SF-36	Emotional health SF-36	Social functioning SF-36	Bodily pain SF-36	General health SF-36
Comb hair	-0.49	-0.27	-0.11^a^	-0.27	-0.17	-0.24	-0.39	-0.34
Walk for 20 minutes	-0.78	-0.43	-0.21	-0.25	-0.24	-0.34	-0.55	-0.41
Prepare a meal	-0.62	-0.45	-0.30	-0.35	-0.29	-0.46	-0.54	-0.45
Clean floors	-0.67	-0.51	-0.33	-0.28	-0.20	-0.43	-0.50	-0.47
Carry a bag of groceries	-0.70	-0.46	-0.23	-0.32	-0.18	-0.36	-0.45	-0.41
Climb a flight of stairs	-0.78	-0.41	-0.19	-0.35	-0.24	-0.32	-0.51	-0.45
Change bed sheets	-0.70	-0.45	-0.23	-0.30	-0.18	-0.34	-0.47	-0.39
Sit for 45 minutes	-0.34	-0.28	-0.07^a^	-0.27	-0.16	-0.18	-0.32	-0.24
Go shopping for groceries	-0.70	-0.47	-0.23	-0.39	-0.26	-0.36	-0.50	-0.46
FIQR function	-0.80	-0.51	-0.26	-0.40	-0.27	-0.41	-0.60	-0.49
Goals	-0.61	-0.54	-0.34	-0.45	-0.35	-0.50	-0.61	-0.48
Overwhelmed	-0.52	-0.42	-0.40	-0.45	-0.49	-0.50	-0.60	-0.46
FIQR overall	-0.60	-0.51	-0.39	-0.48	-0.45	-0.53	-0.64	-0.50
Pain rating	-0.46	-0.42	-0.23	-0.38	-0.24	-0.37	-0.66	-0.40
Energy rating	-0.41	-0.40	-0.26	-0.45	-0.31	-0.32	-0.42	-0.36
Stiffness rating	-0.43	-0.35	-0.16	-0.40	-0.22	-0.28	-0.47	-0.30
Sleep quality	-0.35	-0.27	-0.27	-0.43	-0.33	-0.37	-0.44	-0.41
Depression level	-0.31	-0.25	-0.57	-0.35	-0.73	-0.54	-0.44	-0.41
Memory problems	-0.39	-0.32	-0.26	-0.45	-0.38	-0.35	-0.45	-0.39
Anxiety level	-0.26	-0.26	-0.47	-0.34	-0.63	-0.55	-0.47	-0.40
Tenderness level	-0.38	-0.28	-0.24	-0.31	-0.28	-0.33	-0.47	-0.26
Balance problems	-0.49	-0.33	-0.19	-0.35	-0.25	-0.26	-0.50	-0.39
Environmental sensitivity	-0.34	-0.26	-0.12^a^	-0.26	-0.19	-0.25	-0.30	-0.34
FIQR symptoms	-0.56	-0.46	-0.43	-0.55	-0.54	-0.55	-0.66	-0.54
FIQR total	-0.71	-0.54	-0.39	-0.53	-0.46	-0.54	-0.68	-0.57

^a^These three correlations under 'emotional role' were not significant. All other correlations were significant: *r *≥ 0.15, *P *< 0.05; *r *≥ 0.18, *P *< 0.01; and *r *≥ 0.22, *P *< 0.001. Note: all correlations are negative as the 36-Item Short Form Health Survey (SF-36) scoring has a direction opposite to that of the Revised Fibromyalgia Impact Questionnaire (FIQR).

**Figure 2:**
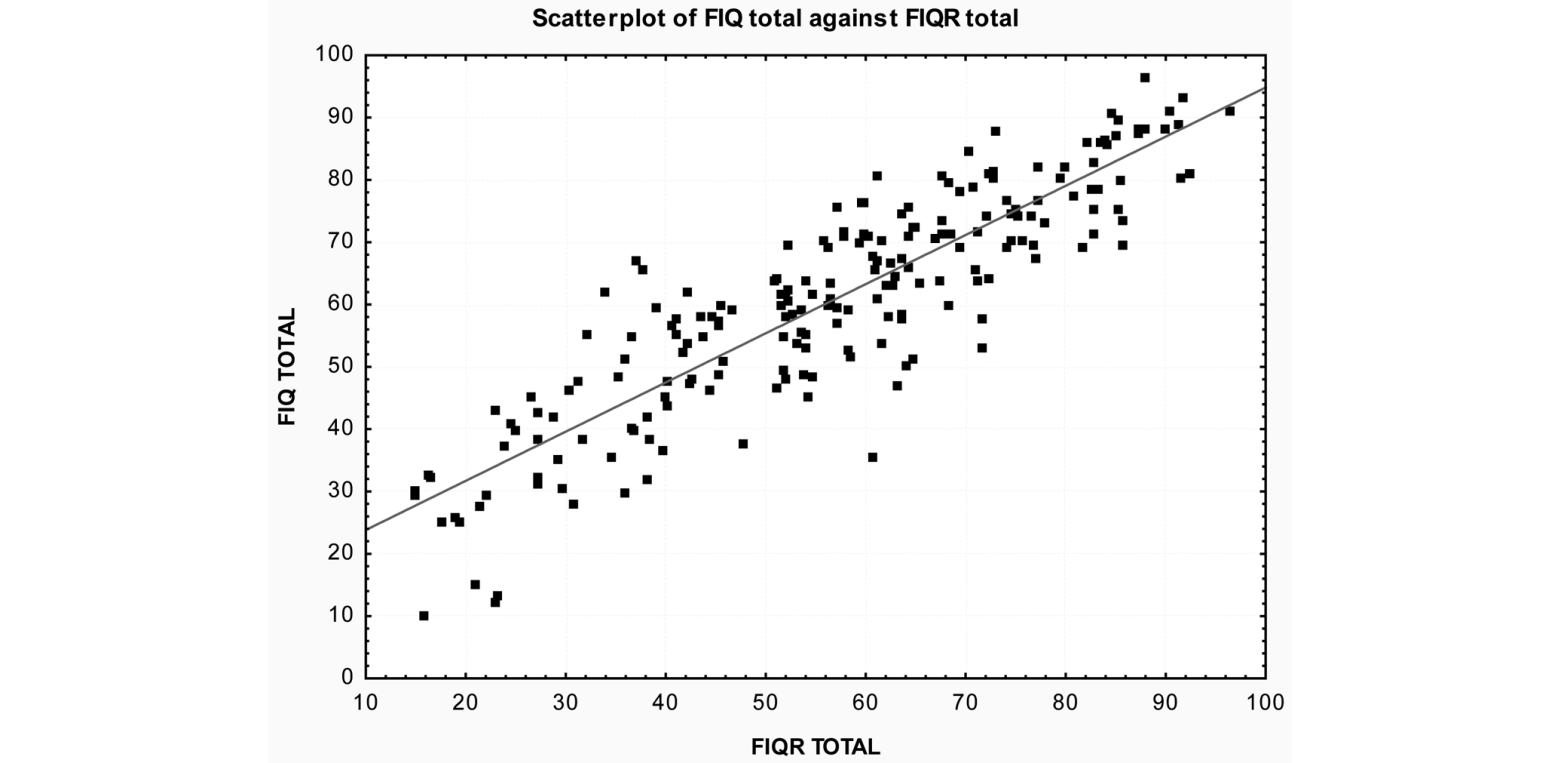
A scatterplot of the total score for the Revised Fibromyalgia Impact Questionnaire (FIQR) and the Fibromyalgia Impact Questionnaire (FIQ) on all 202 fibromyalgia subjects (*r *= 0.88, *P *< 0.001).

As the original FIQ is extensively validated through its use in over 250 studies, we compared FIQR with the original FIQ. The total score of the FIQR in FM patients was 56.58 ± 20 (range 15 to 97), whereas the total score for the FIQ was 60.56 ± 18.0 (range 10 to 96). While this difference is statistically significant (*P *= 0.03), the strong correlation of 0.88 (*P *< 0.001) between the FIQR and FIQ indicates that patients' relative standings on the two scales are very similar. This is indicated by the reasonable correspondence between FM participants' scores on the FIQR and FIQ in the scatterplot (Figure 2). There was a strong correlation of the three domains of the FIQR plus pain with the corresponding domains of the FIQ (Table 7). The correlations along the diagonal (*r *= 0.69 to 0.88), which represents the relation between *corresponding *constructs on the new and old scales, are higher than the correlations between *different *constructs (*r *= 0.46 to 0.75), those below and above the diagonal. This provides further support for the 'domain' structure of the FIQR.

**Table 7:** Pearson correlations of major components of the Fibromyalgia Impact Questionnaire with those of the Revised Fibromyalgia Impact Questionnaire

	FIQ function	FIQ overall	FIQ pain	FIQ symptoms
FIQR function	0.69	0.62	0.59	0.65
FIQR overall	0.56	0.69	0.60	0.75
FIQR pain	0.46	0.55	0.75	0.66
FIQR symptoms	0.54	0.65	0.66	0.88

All correlations were significant at *P *< 0.001. FIQ, Fibromyalgia Impact Questionnaire; FIQR, Revised Fibromyalgia Impact Questionnaire.

Multiple regression analysis was used to determine how well the three FIQR domain scores predicted the eight SF-36 domains (Table 8). In contrast to the correlational analyses presented in Table 6, multiple regression analysis identified both the combined and unique variance that predictor variables contribute to an SF-36 subscale. The three FIQR domains (function, overall impact, and symptoms) were entered simultaneously into the regression equation to predict how much variance in SF-36 domains could be explained by FIQR components. Column 1 shows the multiple R and combined variance. Columns 2, 3, and 4 identify the FIQR components that uniquely predict SF-36 domains. It is seen that all three FIQR domains contributed collectively and uniquely to all SF-36 domains. Column 1 shows multiple correlations ranging from 0.45 to 0.80, with FIQR components collectively explaining 62% of SF-36 physical functioning, 48% of SF-36 pain, and 30% of SF-36 vitality. Columns 2, 3, and 4 show that the FIQR domains predicted *unique *variance in SF-36 domains, providing good discriminant validity. Overall, FIQR domains predicted unique variance in 15 of 24 instances, providing substantial justification for separating the FIQR into three domains. Notably, FIQR function strongly predicted SF-36 physical functioning and role limitation due to physical health (column 2) whereas FIQR symptoms predicted each of the other six remaining SF-36 domains, including SF-36 pain, vitality, emotional health, well-being, and social functioning (column 4). The FIQR 'overall impact' domain, which assesses whether FM prevented goals from being accomplished and whether the patient felt overwhelmed, predicted SF-36 subscales of pain, role limitations due to physical health, emotional well-being, and social functioning; it did not predict physical functioning, general health, vitality, or role limitation due to emotional health. Importantly, each of the three FIQR domains contributed uniquely to the SF-36 pain subscale, illustrating that each of the FIQR domains is relevant to the assessment of pain in FM. In sum, the FIQR, conceptualized around three linked domains, showed both convergent and discriminant validity in predicting SF-36 subscales.

**Table 8:** Multiple regression analysis showing how the three domains of the Revised Fibromyalgia Impact Questionnaire predict subscales of the 36-Item Short Form Health Survey

SF-36 subscales (dependent variable)	R and R^2 ^predicted by combined FIQR domains	FIQR function	FIQR overall impact	FIQR symptoms
Physical functioning	R = 0.80^a^	β = -0.803^a^	β = -0.005	β = 0.015
	R^2 ^= 0.62	pr = -0.641^a^	pr = -0.004	pr = 0.014
Role limitation due to physical health	R = 0.55^a^	β = -0.270^b^	β = -0.261^c^	β = -0.058
	R^2 ^= 0.29	pr = -0.200^b^	pr = -0.167^c^	pr = -0.040
Role limitation due to emotional health	R = 0.45^a^	β = 0.170	β = -0.234^c^	β = -0.362^d^
	R^2 ^= 0.19	pr = 0.120	pr = -0.140^c^	pr = -0.237^d^
Energy/Fatigue	R = 0.55^a^	β = 0.029	β = -0.133	β = -0.465^a^
	R^2 ^= 0.30	pr = 0.022	pr = -0.080	pr = -0.312^a^
Emotional well-being	R = 0.58^a^	β = 0.308^d^	β = -0.210^c^	β = -0.593^a^
	R^2 ^= 0.32	pr = 0.231^d^	pr = -0.137^c^	pr = -0.392^a^
Social functioning	R = 0.57^a^	β = 0.066	β = -0.287^b^	β = -0.369^d^
	R^2 ^= 0.32	pr = 0.050	pr = -0.186^b^	pr = -0.256^d^
Pain	R = 0.70^a^	β = -0.175^c^	β = -0.219^c^	β = -0.362^a^
	R^2 ^= 0.48	pr = -0.0152^c^	pr = -0.163^c^	pr = -0.285^a^
General health	R = 0.57^a^	β = -0.185^c^	β = -0.085	β = -0.347^d^
	R^2 ^= 0.31	pr = -0.140^c^	pr = -0.056	pr = -0.241^d^

^a^*P *< 0.0001; ^b^*P *< 0.01; ^c^*P *< 0.05; ^d^*P *< 0.001. First column: adjusted R-square (R^2^) × 100 indicates the total variance in 36-Item Short Form Health Survey (SF-36) subscale accounted for by the common and unique variance in Revised Fibromyalgia Impact Questionnaire (FIQR) function, overall, and symptom domains taken together. Multiple regression (R) indicates the size of correlation between three FIQR domains as predictors taken together with the SF-36 subscale as criterion. Columns 2, 3, and 4 present the standardized regression (β) coefficients, which represent the unique contribution of the predictor variable, and the partial correlation (pr) coefficients, which represent the correlation for the one (FIQR) domain with the SF-36 subscale after controlling for the other two (FIQR) domains.

**Figure 3:**
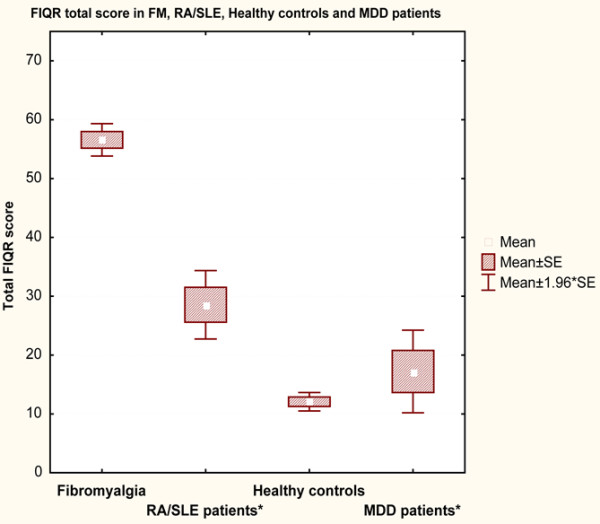
The total Revised Fibromyalgia Impact Questionnaire (FIQR) scores of the 202 fibromyalgia (FM) patients compared with the scores for the 213 healthy controls, 51 patients with rheumatoid arthritis (RA) or systemic lupus erythematosus (SLE), and 11 patients with major depression. *Note: a concomitant diagnosis of FM was an exclusion criterion for inclusion of RA/SLE and major depressive disorder (MDD) subjects. SE, standard error.

Discriminant validity was also evaluated by comparing the FIQR total scores in FM patients (56.6 ± 19.9, 95% CI 53.8, 59.4) with the scores in healthy controls (12.1 ± 11.6, 95% CI 10.5, 13.6), patients being treated for RA or SLE (28.6 ± 21.2, 95% CI 22.6, 34.5), and patients under treatment for MDD (17.3 ± 11.8, 95% CI 9.3, 25.2) (Figure 3). As noted in Materials and methods, the FIQR for these three groups substituted 'health issues' for 'fibromyalgia'. These four total FIQR scores were significantly different: F(3,473) = 247.94 (*P *< 0.001). The FM FIQR total score was significantly higher than in the three other groups (Tukey HSD test *P *< 0.001 for all three comparisons). The FIQR in the RA/SLE group (28.6 ± 21.2) was significantly higher than in the healthy group (12.1 ± 11.6) (*P *< 0.02). The MDD total FIQR score (17.3 ± 12) did not differ from the healthy and RA/SLE groups.

**Figure 4:**
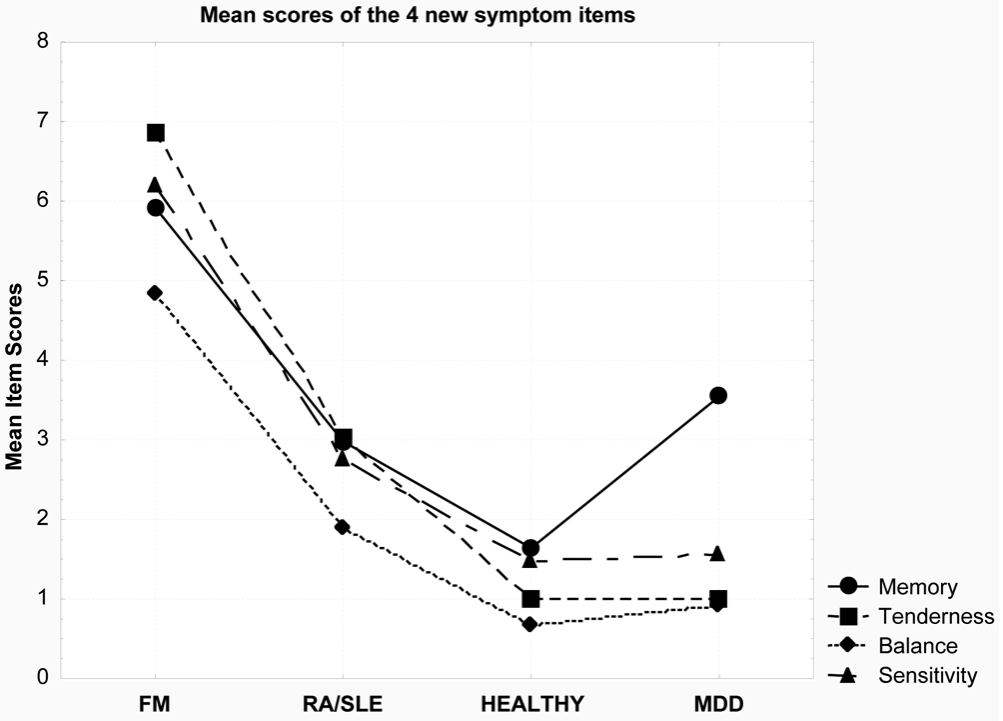
A plot of the mean scores for the four new symptoms added to the Revised Fibromyalgia Impact Questionnaire (memory, tenderness, balance, and environmental sensitivity) against each of the four groups: fibromyalgia (FM), rheumatoid arthritis/systemic lupus erythematosus (RA/SLE), healthy, and major depressive disorder (MDD).

A similar analysis was conducted to determine whether the FM group differed from the other three groups on the four new FIQR symptoms (memory, tenderness, balance, and sensitivity). If the four new symptoms reflect FM impact, then group differences on these symptoms should emerge, providing evidence for the construct validity for the syndrome. Figure 4, which presents the means of all four groups with respect to each of the four new symptoms, shows that the four groups discriminated between the four subject groups (Wilks lambda = 0.33, RaoR(12, 1,243) = 53.86, *P *< 0.001), with the FM patients scoring substantially higher than the other three groups. Additionally, the FM group scored substantially higher than all three other groups on all four symptoms (*P *< 0.001), with the singular exception of the comparison with the MDD group on memory (*P *< 0.07). Figure 4 also illustrates the significant mean differences on these four symptoms in the FM group (highest to lowest rankings: tenderness, sensitivity, memory, and balance). Tenderness, the most problematic symptom for FM patients, was significantly higher than both sensitivity (*P *< 0.004) and memory (*P *< 0.001). Balance, the least problematic, was significantly lower than both sensitivity (*P *< 0.001) and memory (*P *< 0.001). Despite these differences, which contribute to the overall individual differences in the FIQR total scores, the item-FIQR total correlations for the four new symptom items (*r *= 0.56, 0.57, 0.69, and 0.65) were similar, indicating that they are of nearly equal relevance for defining the FM syndrome. The RA/SLE group had significantly higher scores for the four new symptoms than the healthy controls (*P *< 0.001), thus justifying the inclusion of RA/SLE as an intermediate group.

## Discussion

We describe and validate a revised version of the FIQ: the FIQR. This version was developed in an attempt to correct some of the problems in the wording, omissions, concepts, and scoring of the original FIQ [1,2]. There are several modifications of the FIQ which have been incorporated into the FIQR, while retaining the basic domain structure in terms of function, overall impact, and severity of symptoms that are characteristic of FM (Table 1). Each of the three FIQR domains was highly correlated with the total FIQR score and predicted unique variance in SF-36 domains, providing good evidence for discriminant validity. The mean total score of the FIQR was approximately 4 points lower than the mean FIQ total score; we attribute this to the change of the weighting in the scoring algorithm.

The first domain, function, in the FIQR has been reduced to 9 questions from the original 11 questions and now has a weighting of 30% of the total score, as opposed to 10% in the FIQ, to reflect the relative importance of function in assessing the impact of FM. The specific questions in the function domain have been modified to reflect a better balance between large-muscle activities of the upper and lower limbs and have less gender and ethnicity bias than the FIQ. Importantly, the FIQR function domain was most highly correlated with the SF-36 physical functioning subscale. In a multiple regression model, FIQR function most strongly and uniquely (that is, after removing shared and unique variance with the other domains) related to SF-36 physical functioning (Table 8).

The second domain, overall impact, has been completely revised to reflect two subdomains, namely the overall impact of FM on functional ability and the overall impact of FM on the perception of reduced function (Figure 1). The FIQR overall impact domain was most highly correlated with the SF-36 subscales of physical functioning and pain (Table 6). In a multiple regression analysis, the overall impact domain was most specifically associated with the SF-36 subscales of social functioning, role limitation due to physical health, and emotional well-being. There was a moderately good correlation of the FIQR overall impact domain with the FIQ overall (Table 8). The weighting of this FIQR domain remains the same as the FIQ (that is, 20% of the total score).

The third domain, symptoms, retains the original questions in the FIQ regarding pain, stiffness, lack of restorative sleep, poor energy, anxiety, and depression and adds four additional questions relating to tenderness, memory, balance, and environmental sensitivity (Figure 1). These questions were added in light of ongoing experience with OMERACT patient delphi exercises [16], ICF guidelines [8], patient surveys [9], and clinical testing [17]. The weighting for this FIQR domain is 50% of the total score as opposed to 70% in the FIQ. These four new symptom questions all had strong correlations with the total FIQR score, and each provided discriminant validity between the healthy controls, the patients with RA/SLE, and the patients with MDD. Furthermore, all four items discriminated between the four subject groups, with the FM patients scoring substantially and significantly higher than the other three groups. The scores on memory were similar in the FM group and the MDD group, probably an expression of the well-documented memory problems associated with depressive illness [18,19]. It is interesting to note that, although the FM and MDD groups had similar scores on depression and anxiety, the FM patients had distinctively higher scores on tenderness, environmental sensitivity, and balance. This was also the case when comparing the FM with the RA/SLE group. Thus, the FM patients displayed distinctive responses to these four new questions.

Although all of the FIQR symptom items and the FIQR symptom domain were correlated with all of the SF-36 subscales, multiple regression analysis indicated that the symptom domain provided more unique variance to six of the SF-36 subscales (role limitation due to emotional health, vitality, emotional well-being, social functioning, pain, and general health) than did the other two FIQR domains. Coupled with the function domain uniquely predicting SF-36 physical functioning, this analysis provides substantial discriminant validity for the domain structure of the FIQR in relation to the SF-36. Each of the 21 FIQR questions can be related to relevant outcomes as specified by the ICF guidelines [20] (Table 9). Prodinger and colleagues [5] reported a closer ICF correspondence between the FIQ and SF-36 compared with 14 other general health instruments; thus, the current finding showing a strong relation between FIQR and the SF-36 provides further confirmation of the content validity of the FIQR. However, it is worth noting that substantial FM variance (column 1 of Table 8) is *not *captured by the SF-36, suggesting that the FIQR is measuring unique variance that is distinctive and specific to the FM syndrome.

**Table 9:** Guidelines of the International Classification of Functioning, Disability, and Health applied to each of the 21 questions of the Revised Fibromyalgia Impact Questionnaire

FIQR question	Nearest ICF category
Brush or comb your hair	d5202 (caring for hair)
Walk continuously for 20 minutes	d4500 (walking short distances)
Prepare a homemade meal	d6300 (preparing a simple meal)
Vacuum, scrub, or sweep floors	d6402 (cleaning living area)
Lift and carry a bag full of groceries	d430 (lifting and carrying an object)
Climb one flight of stairs	d4551 (climbing)
Change bed sheets	d6408 (doing housework)
Sit in a chair for 45 minutes	d4153 (maintaining a sitting position)
Go shopping for groceries	d6200 (shopping)
Fibromyalgia prevented me from accomplishing goals for the week	d2302 (completing daily routine)
I was completely overwhelmed by my fibromyalgia symptoms	b1809 (experience of self and time function)
Please rate your level of pain	b2800 (generalized pain)
Please rate your level of energy	b1300 (energy level)
Please rate your level of stiffness	b7800 (sensation of muscle stiffness)
Please rate the quality of your sleep	b1343 (quality of sleep)
Please rate your level of depression	b152 (emotional function)
Please rate your level of memory problems	b144 (memory function)
Please rate your level of anxiety	b1470 (psychomotor function)
Please rate your level of tenderness to touch	b2702 (sensitivity to pressure)
Please rate your level of balance problems	b2531 (vestibular function of balance)
Please rate your level of sensitivity to loud noises, bright lights, odors, and cold	b2708 (sensory function related to temperature and other stimuli)

FIQR, Revised Fibromyalgia Impact Questionnaire; ICF, International Classification of Functioning, Disability, and Health.

The use of a numeric rating scale using 11 boxes, scored 0 to 10, in the FIQR as opposed to the combination of Likert and VAS scaling in the FIQ did not result in significant differences in the total scores in the focus group analysis (Table 2). Furthermore, there was excellent correlation between the paper version with VAS scoring (FIQ-P VAS), the paper version using the 0-to-10 numeric rating (FIQ-P), and the online FIQR using the 0-to-10 numeric rating (FIQR-OL) (Table 2). The use of the numeric rating considerably simplifies the scoring algorithm for the FIQR and obviates the need to use a ruler to measure VAS scores. Furthermore, the use of the numeric rating scoring greatly simplifies the conversion of a paper version of the FIQR to an online version, as done in this study. The paper version of the FIQR took approximately half the time to complete compared with the FIQ. On the other hand, the SF-36 took nearly four times as long to complete as the FIQR. Our favorable experience with the use of a numeric rating scale compared with continuous VAS scoring reflects the experience of several other groups [21-23] and is in line with the recommendations of the Initiative on Methods, Measurement, and Pain Assessment in Clinical Trials (IMMPACT) [24]. This simplification and greater efficiency should make the FIQR easier to use by researchers and physicians. The FIQR shows good ability to discriminate between FM patients and patients with RA, SLE, and MDD who do not have concomitant FM.

There are several limitations to the interpretation of this study. The testing was done entirely online; thus, it is not possible to equate these results with a paper version of the FIQR. However, a comparison of the paper and online versions was completed by the focus group and showed no significant differences between the two methods. No test-retest reliability was performed on the online participants, but again the limited information from the focus group suggested good test-retest reliability. Only 12 males completed the questionnaires; thus, their FIQR scores cannot be considered representative of a large male population. We were able to recruit only 11 subjects with MDD who did not have FM; thus, the validity of comparisons associated with MDD may be limited. We could not estimate the sensitivity to change of the FIQR or the minimal clinical important difference (MCID) for scoring the FIQR, as has been done for the FIQ [25]; these analyses will have to await the use of the FIQR in a large clinical trial. Given that there are currently no generally accepted objective measures of FM severity, validated questionnaires measuring patients' subjective responses will continue to be important. In this respect, we hope this revised version of the FIQ will be useful to both researchers and clinicians.

## Conclusions

A revised version of the FIQ, called the FIQR, is described herein. The FIQR has sound psychometric properties, discriminates between FM patients and patients with RA, SLE, and MDD, takes just over 1 minute to complete, is easy to score, and can be used in online surveys. The FIQR has a good correlation with the original FIQ, thus providing the ability to compare the results of studies using the older version with studies using the revised version.

## Abbreviations

ACR: American College of Rheumatology; ANOVA: analysis of variance; CI: confidence interval; FIQ: Fibromyalgia Impact Questionnaire; FIQ-OL: an online version of the Fibromyalgia Impact Questionnaire; FIQ-P: the original paper version of the Fibromyalgia Impact Questionnaire; FIQR: Revised Fibromyalgia Impact Questionnaire; FIQR-OL: an online version of the Revised Fibromyalgia Impact Questionnaire; FIQR-P: a paper version of the Revised Fibromyalgia Impact Questionnaire using 11 boxes scaled 0 to 10; FIQR-P VAS: a paper version of the Revised Fibromyalgia Impact Questionnaire using a 100-mm visual analog scale scoring instead of 11 boxes; FM: fibromyalgia; HSD: honestly significantly differences; ICF: International Classification of Functioning, Disability, and Health; MDD: major depressive disorder; OMERACT: Outcome Measures in Rheumatology; RA: rheumatoid arthritis; SF-36: 36-Item Short Form Health Survey; SLE: systemic lupus erythematosus; VAS: visual analog scale.

## Competing interests

The authors declare that they have no competing interests.

## Authors' contributions

RMB conceived of and guided the study, directed the focus group, provided the algorithms for Survey Monkey, downloaded and analyzed the results, and drafted the manuscript. RF provided statistical analyses and was involved in the writing and editing of the manuscript. KDJ assisted in the focus group and was involved in the writing and editing of the manuscript. RW assisted in the focus group and was responsible for checking the integrity of the databases. BKH recruited the patients with RA from his clinical practice and reviewed the manuscript. RLR recruited the patients with MDD from her clinical practice and helped in editing the manuscript. All authors read and approved the final manuscript.

## Supplementary Material

Additional data file 1 A PDF that provides the 2 page version of the FIQR that is suitable for printing. Click here for file

Additional data file 2 A PDF that provides the 2 page version of the FIQR that was used in the non-fibromyalgia patients; this questionnaire does not use the word 'fibromyalgia' and is termed the Symptom Impact Questionnaire (SIQR). Click here for file
