# Supplementary material for: The Revised Fibromyalgia Impact Questionnaire (FIQR): validation and psychometric properties
Source: Arthritis Res Ther. 2009 Aug 10;11(4):R120. doi: 10.1186/ar2783 (PMC2745803; doi:10.1186/ar2783)
Supplement: Additional data file 1 — A PDF that provides the 2 page version of the FIQR that is suitable for printing. [file ar2783-S1.pdf]

REVISED FIBROMYALGIA IMPACT QUESTIONNAIRE (FIQR)

Last Name: **First Name:** **Age:**

Directions: For each question, place an "**X**" in the box that best indicates how much your fibromyalgia made it difficult to do each of the following activities during the past 7 days

Brush or comb your hair	**No difficulty** ☐ ☐ ☐ ☐ ☐ ☐ ☐ ☐ ☐ ☐ ☐ **Very difficult**
Walk continuously for 20 minutes	**No difficulty** ☐ ☐ ☐ ☐ ☐ ☐ ☐ ☐ ☐ ☐ ☐ **Very difficult**
Prepare a homemade meal	**No difficulty** ☐ ☐ ☐ ☐ ☐ ☐ ☐ ☐ ☐ ☐ ☐ **Very difficult**
Vacuum, scrub or sweep floors	**No difficulty** ☐ ☐ ☐ ☐ ☐ ☐ ☐ ☐ ☐ ☐ ☐ **Very difficult**
Lift and carry a bag full of groceries	**No difficulty** ☐ ☐ ☐ ☐ ☐ ☐ ☐ ☐ ☐ ☐ ☐ **Very difficult**
Climb one flight of stairs	**No difficulty** ☐ ☐ ☐ ☐ ☐ ☐ ☐ ☐ ☐ ☐ ☐ **Very difficult**
Change bed sheets	**No difficulty** ☐ ☐ ☐ ☐ ☐ ☐ ☐ ☐ ☐ ☐ ☐ **Very difficult**
Sit in a chair for 45 minutes	**No difficulty** ☐ ☐ ☐ ☐ ☐ ☐ ☐ ☐ ☐ ☐ ☐ **Very difficult**
Go shopping for groceries	**No difficulty** ☐ ☐ ☐ ☐ ☐ ☐ ☐ ☐ ☐ ☐ ☐ **Very difficult**

Function sub-total ☐
(for internal use only)

Directions: For each question, check the <u>one</u> box that best describes the overall impact of your fibromyalgia over the last 7 days:

Fibromyalgia prevented me from accomplishing goals for the week	**Never** ☐ ☐ ☐ ☐ ☐ ☐ ☐ ☐ ☐ ☐ ☐ **Always**
I was completely overwhelmed by my fibromyalgia symptoms	**Never** ☐ ☐ ☐ ☐ ☐ ☐ ☐ ☐ ☐ ☐ ☐ **Always**

Overall Impact sub-total ☐
(for internal use only)

Directions: For each of the following 10 questions, select the one circle that best indicates the intensity of your fibromyalgia symptoms over the past 7 days

Please rate your level of pain	**No pain** ☐ ☐ ☐ ☐ ☐ ☐ ☐ ☐ ☐ ☐ ☐ **Unbearable pain**
Please rate your level of energy	**Lots of energy** ☐ ☐ ☐ ☐ ☐ ☐ ☐ ☐ ☐ ☐ ☐ **No energy**
Please rate your level of stiffness	**No stiffness** ☐ ☐ ☐ ☐ ☐ ☐ ☐ ☐ ☐ ☐ ☐ **Severe stiffness**
Please rate the quality of your sleep	**Awoke well rested** ☐ ☐ ☐ ☐ ☐ ☐ ☐ ☐ ☐ ☐ ☐ **Awoke very tired**
Please rate your level of depression	**No depression** ☐ ☐ ☐ ☐ ☐ ☐ ☐ ☐ ☐ ☐ ☐ **Very depressed**
Please rate your level of memory problems	**Good memory** ☐ ☐ ☐ ☐ ☐ ☐ ☐ ☐ ☐ ☐ ☐ **Very poor memory**
Please rate your level of anxiety	**Not anxious** ☐ ☐ ☐ ☐ ☐ ☐ ☐ ☐ ☐ ☐ ☐ **Very anxious**
Please rate your level of tenderness to touch	**No tenderness** ☐ ☐ ☐ ☐ ☐ ☐ ☐ ☐ ☐ ☐ ☐ **Very tender**
Please rate your level of balance problems	**No imbalance** ☐ ☐ ☐ ☐ ☐ ☐ ☐ ☐ ☐ ☐ ☐ **Severe imbalance**
Please rate your level of sensitivity to loud noises, bright lights, odors and cold	**No sensitivity** ☐ ☐ ☐ ☐ ☐ ☐ ☐ ☐ ☐ ☐ ☐ **Extreme sensitivity**

Symptom sub-total ☐
(for internal use only)

FIQR TOTAL SCORE ☐
(for internal use only)
