# Supplementary material for: The Revised Fibromyalgia Impact Questionnaire (FIQR): validation and psychometric properties
Source: Arthritis Res Ther. 2009 Aug 10;11(4):R120. doi: 10.1186/ar2783 (PMC2745803; doi:10.1186/ar2783)
Supplement: Additional data file 2 — A PDF that provides the 2 page version of the FIQR that was used in the non-fibromyalgia patients; this questionnaire does not use the word 'fibromyalgia' and is termed the Symptom Impact Questionnaire (SIQR). [file ar2783-S2.pdf]

SYMPTOM IMPACT QUESTIONNAIRE (SIQR)

Last Name: **First Name:** **Age:**

Directions: For each question, place an "**X**" in the box that best indicates how much difficulty you have experienced in doing the following activities during the past 7 days.

Brush or comb your hair	**No difficulty**	☐ ☐ ☐ ☐ ☐ ☐ ☐ ☐ ☐ ☐ ☐	**Very difficult**
Walk continuously for 20 minutes	**No difficulty**	☐ ☐ ☐ ☐ ☐ ☐ ☐ ☐ ☐ ☐ ☐	**Very difficult**
Prepare a homemade meal	**No difficulty**	☐ ☐ ☐ ☐ ☐ ☐ ☐ ☐ ☐ ☐ ☐	**Very difficult**
Vacuum, scrub or sweep floors	**No difficulty**	☐ ☐ ☐ ☐ ☐ ☐ ☐ ☐ ☐ ☐ ☐	**Very difficult**
Lift and carry a bag full of groceries	**No difficulty**	☐ ☐ ☐ ☐ ☐ ☐ ☐ ☐ ☐ ☐ ☐	**Very difficult**
Climb one flight of stairs	**No difficulty**	☐ ☐ ☐ ☐ ☐ ☐ ☐ ☐ ☐ ☐ ☐	**Very difficult**
Change bed sheets	**No difficulty**	☐ ☐ ☐ ☐ ☐ ☐ ☐ ☐ ☐ ☐ ☐	**Very difficult**
Sit in a chair for 45 minutes	**No difficulty**	☐ ☐ ☐ ☐ ☐ ☐ ☐ ☐ ☐ ☐ ☐	**Very difficult**
Go shopping for groceries	**No difficulty**	☐ ☐ ☐ ☐ ☐ ☐ ☐ ☐ ☐ ☐ ☐	**Very difficult**

Sub-total ***(for internal use only)*** ☐

Directions: For each question, check the one box that best describes the overall impact of any medical problems over the last 7 days:

Was able to accomplish most set goals for week	**Always**	☐ ☐ ☐ ☐ ☐ ☐ ☐ ☐ ☐ ☐ ☐	**Never**
Was overwhelmed by symptoms	**Never**	☐ ☐ ☐ ☐ ☐ ☐ ☐ ☐ ☐ ☐ ☐	**Always**

Sub-total ***(for internal use only)*** ☐

Directions: For each question, check the <u>one</u> box that best indicates the intensity, during the past 7 days, of the following common symptoms.

How severe has your pain been?	**No pain**	☐ ☐ ☐ ☐ ☐ ☐ ☐ ☐ ☐ ☐ ☐	**Unbearable pain**
How much were you fatigued or lacking in energy?	**Lots of energy**	☐ ☐ ☐ ☐ ☐ ☐ ☐ ☐ ☐ ☐ ☐	**No energy**
How much has stiffness bothered you?	**No stiffness**	☐ ☐ ☐ ☐ ☐ ☐ ☐ ☐ ☐ ☐ ☐	**Severe stiffness**
How did you feel on awakening in the morning?	**Well rested**	☐ ☐ ☐ ☐ ☐ ☐ ☐ ☐ ☐ ☐ ☐	**Very tired**
How depressed or blue have you felt?	**No depression**	☐ ☐ ☐ ☐ ☐ ☐ ☐ ☐ ☐ ☐ ☐	**Very depressed**
How much has memory and / or concentration been a problem?	**Not a problem**	☐ ☐ ☐ ☐ ☐ ☐ ☐ ☐ ☐ ☐ ☐	**Severe problem**
How stressed or anxious have you felt?	**Not anxious**	☐ ☐ ☐ ☐ ☐ ☐ ☐ ☐ ☐ ☐ ☐	**Very anxious**
How much tenderness to touch have you felt?	**No tenderness**	☐ ☐ ☐ ☐ ☐ ☐ ☐ ☐ ☐ ☐ ☐	**Very tender**
How much has balance and coordination been a problem *(e.g. falling)?*	**No imbalance**	☐ ☐ ☐ ☐ ☐ ☐ ☐ ☐ ☐ ☐ ☐	**Severe imbalance**
How much environmental sensitivity have you had *(e.g. noises, lights, odors, cold)*?	**No sensitivity**	☐ ☐ ☐ ☐ ☐ ☐ ☐ ☐ ☐ ☐ ☐	**Extreme sensitivity**

Sub-total ***(for internal use only)*** ☐

SIQR TOTAL ***(for internal use only)*** ☐
